# Psychometric properties of the Chalder Fatigue Scale revisited: an exploratory structural equation modeling approach

**DOI:** 10.1007/s11136-015-0944-4

**Published:** 2015-02-17

**Authors:** Ted C. T. Fong, Jessie S. M. Chan, Cecilia L. W. Chan, Rainbow T. H. Ho, Eric T. C. Ziea, Vivian C. W. Wong, Bacon F. L. Ng, S. M. Ng

**Affiliations:** 1 Centre on Behavioral Health, The University of Hong Kong, Hong Kong, China; 2 Department of Social Work and Social Administration, Jockey Club Tower, The Centennial Campus, The University of Hong Kong, Pokfulam, Hong Kong, China; 3 Chinese Medicine Department, Hong Kong Hospital Authority, Hong Kong, China

**Keywords:** Chinese, Chronic fatigue, Convergent validity, Cross-loadings, Factor structure

## Abstract

**Objective:**

Previous validation studies of the Chalder Fatigue Scale (CFS) suffer methodological shortcomings. The present study aimed to re-evaluate its psychometric properties using exploratory structural equation modeling (ESEM).

**Methods:**

A Chinese sample of 1259 community-dwelling residents completed the 11-item Chinese CFS and a variety of health measures (anxiety, depression, exhaustion, sleep disturbance, and quality of life). In addition to traditional confirmatory factor analysis, ESEM was performed to assess the fit of two- and three-factor models using robust maximum likelihood estimation and oblique geomin rotation. Convergent validity of the CFS was examined via associations with five covariates (gender, age, exercise, perceived health, and life event) and the health measures in the ESEM model.

**Results:**

The ESEM models displayed a superior fit to confirmatory factor models. The three-factor ESEM model showed a satisfactory model fit to the data but not for the two-factor model. The three factors were physical fatigue (three items, *α* = .800), low energy (four items, *α* = .821), and mental fatigue (four items, *α* = .861). The factors exhibited convergent validity with the model covariates and health measures.

**Conclusion:**

The results demonstrate the satisfactory reliability and convergent validity for the three-factor structure of the CFS as a valid measure of fatigue symptoms in the general population. Future psychometric studies could adopt the ESEM approach as a practical alternative to traditional confirmatory factor analysis.

## Introduction

Chronic fatigue is a symptom commonly reported by patients in primary care practice and by the general population, with prevalence of 11.3 % among British primary care patients [1] and of 10.7 % among the general population of Hong Kong [2]. Patients with relapsing and unexplained fatigue that persists for at least 6 months are said to suffer from chronic fatigue syndrome. This debilitating syndrome is associated with significant disability in the functioning capacity of the cognitive and psychosocial domains [3]. The 11-item Chalder Fatigue Scale (CFS) [4, 5] was developed as an assessment tool for fatigue in both general and clinical populations [6, 7]. The scale has shown adequate degrees of reliability and convergent validity [5, 8, 9].

Regarding the factor structure of the CFS, a two-factor structure was originally proposed [4]. Despite some empirical support for the two-factor structure [8, 10], previous validation studies of the CFS suffer methodological shortcomings. First, most of these studies adopted the outdated principal component analysis and varimax rotation approaches. Principal component analysis does not distinguish shared variance from unique variance [11] and is a biased estimator in factor analysis [12]. The unrealistic orthogonal factors resulting from varimax rotation likely lead to distorted factor structures [12]. The Kaiser’s criterion of retaining factors with eigenvalues that exceed one is known to be unreliable and biased. The frequent use of these outdated approaches diminishes the credibility of these results on the factor structure of the CFS.

Second, Wong and Fielding [13] applied confirmatory factor analysis (CFA) to evaluate the factor structure of the CFS in a Chinese sample. They compared the fit of a two-factor correlated model with a two-factor model with a second-order factor (see their Fig. 1, p. 91). Although they claimed to successfully replicate the original two-factor structure by showing a superior model fit for the latter model, the second-order factor model with only two first-order factors was actually statistically unidentified and addition of a second-order factor should not result in a decrease in model Chi-square. It remains open to question whether their findings replicated the original two-factor model or provided evidence in support of a three-factor model.

Given the methodological limitations of the existing validation studies, there is a clear need for systematic psychometric analysis on this widely used scale. Traditional CFA has been criticized for being overly restrictive in fixing all cross-loadings to zero [14]. The over-restriction could contribute to a lack of model fit and inflated factor correlations in CFA models. Exploratory factor analysis estimates the cross-loadings and results in more realistic factor structure. However, unlike CFA, exploratory factor analysis does not accommodate the use of model covariates or residual correlations. Exploratory structural equation modeling (ESEM) is a newly proposed analytic methodology with substantial modeling flexibility [15, 16]. ESEM allows not only estimations of the cross-loadings and residual correlations, but also incorporation of covariates in the model. The ESEM model has been shown to provide a better model fit and unbiased interfactor correlations [17, 18]. The aim of the study reported herein was to examine the psychometric properties of the CFS in a large Chinese community sample. In particular, we explored the factor structure of the scale using both traditional CFA and ESEM and compared their results.

## Methods

### Sample

This study was based on a convenience sample comprising 1259 community-dwelling residents of Hong Kong (1017 women and 242 men) aged 20–65 (*M* = 43.0, SD = 8.0). The majority of participants was married (62.1 %), worked full time (80.6 %), and had no religion (58.5 %). About half had completed tertiary education (49.8 %), 35.6 % engaged in regular exercise, 24.9 % had experienced a major life event, and 63.6 % reported their perceived health level to be acceptable on a 4-point ordinal scale (1 = *very bad*, 2 = *not good*, 3 = *acceptable*, 4 = *very good*). The participants provided informed consent and completed a self-report online questionnaire on fatigue and health measures. Ethical approval was obtained from the local institutional review board.

### Measures

Fatigue was assessed using the Chinese version of the CFS [13]. This 11-item self-report instrument measures fatigue severity over the past 3 months. The CFS was originally perceived as comprising two subscales that evaluate fatigue in the physical and mental domains. Items are rated on a 4-point Likert scale (0 = *better than usual*, 1 = *no more than usual*, 2 = *worse than usual*, 3 = *much worse than usual*), with higher scores indicating greater fatigue. To evaluate the convergent validity of the CFS, a variety of health measures was used to assess the participants’ levels of anxiety, depression, exhaustion, sleep disturbance, and quality of life.

Anxiety and depression were measured using the 14-item, 4-point Chinese Hospital Anxiety and Depression Scale [19, 20]. Sleep disturbance was measured using the 19-item Chinese Pittsburgh Sleep Quality Index [21], which assesses seven components on a 4-point scale. The total scale scores for anxiety (seven items), depression (seven items), and sleep disturbance ranged from 0 to 21. Higher scores denote worse mental health and greater sleep disturbance. Exhaustion was assessed using the 5-item subscale of the 16-item Chinese Maslach Burnout Inventory [22]. Items are rated on a 7-point scale (ranging from 0 = *never* to 6 = *every day*), with a scoring range of 0–30 and higher scores indicating greater exhaustion. Quality of life was assessed using the 12-item Chinese Short-Form Health Survey [23]. This scale measures health-related quality of life by physical and mental component scores, with a scoring range of 0–100 and higher scores indicating a better quality of life. All of the instruments showed good levels of reliability in the present study, with Cronbach’s *α* = .84, .79, .87, .71, and .78 for anxiety, depression, exhaustion, sleep disturbance, and quality of life, respectively.

### Data analysis

Traditional CFA and recent ESEM were carried out to investigate the factor structure of the CFS using Mplus version 7.2 [24]. The ESEM models used oblique geomin rotation [25] and identified exploratory factors by estimating the factor loadings on all factors within a structural equation modeling framework [15, 16]. To determine the scale’s dimensionality, we estimated and compared the model fit of two CFA models (two- and three-factor CFA models) and three ESEM models (two-, three-, and four-factor ESEM models). Factor loadings >.40 were considered practically significant, and items with no major factor loadings were removed from the model. Model modification was performed based on modification index with reference to standardized expected parameter change [26].

To evaluate the convergent validity of the CFS, we explored the degree to which the derived CFS factors were associated with related constructs (background covariates and concurrent outcomes). Five variables, namely gender, age, exercise, perceived health, and life event, were added to the ESEM model as model covariates. Correlations of the CFS factors with the concurrent health outcomes (anxiety, depression, exhaustion, sleep disturbance, and quality of life) were obtained. Missing data were minimal (<1 %) for all of the study variables in this study.

Model estimations were carried out using the robust maximum likelihood estimator. The reliability of each factor was assessed by Cronbach’s *α*. Model fit was evaluated via the criteria of the following goodness-of-fit indices [27]: comparative fit index (CFI) ≥ .95, Tucker–Lewis index (TLI) ≥ .95, root mean square error of approximation (RMSEA) ≤ .06, and standardized root mean square residual (SRMR) ≤ .08. Model comparison was based on the Bayesian information criterion (BIC) [28], with smaller values denoting a better model. A difference greater than 10 in the BIC indicates a practically significant improvement in model fit.

## Results

### Descriptive statistics

Table 1 shows the descriptive statistics of the CFS items. Overall, the respondents displayed moderate levels of fatigue, with item means ranging from .94 to 2.27 on a scale of 0–3. All 11 of the CFS items exhibited mild degrees of non-normality (magnitude of skewness and kurtosis <1) in the present study. The respondents showed moderate levels of anxiety (*M* = 10.6, SD = 3.9), depression (*M* = 9.1, SD = 3.7), exhaustion (*M* = 16.4, SD = 5.4), and sleep disturbance (*M* = 10.3, SD = 3.7). They showed low levels of quality of life in physical (*M* = 37.3, SD = 7.3) and mental (*M* = 35.1, SD = 9.9) domains.

**Table 1:** Descriptive statistics of the CFS and factor loading matrix of the three-factor ESEM model with geomin rotation

Item		Mean (SD)	Factor		
			Physical fatigue	Low energy	Mental fatigue
1.	Problems with tiredness	2.27 (.68)	.70**	.11	−.01
2.	Rest more	2.22 (.69)	.87**	−.01	−.01
3.	Feel sleepy or drowsy	1.85 (.83)	.44**	.27**	.09
4.	Problems starting things	1.54 (.98)	.01	.72**	.00
5.	Lack energy	1.72 (.97)	−.19	.97**	−.01
6.	Less strength in muscles	1.67 (.97)	.09	.45**	.12**
7.	Feel weak	1.65 (.98)	.02	.66**	.09
8.	Hard to concentrate	1.53 (.93)	−.01	.43**	.46**
9.	Make slips of the tongue	1.04 (.96)	−.01	.02	.86**
10.	Hard to find the correct word	.94 (.95)	−.01	−.03	.88**
11.	Poor memory	1.57 (.99)	.06	.21**	.57**

*SD* standard deviation; factor loadings with magnitude >.40 are bolded; ** *p* < .01

### Factor structure

Table 2 presents the fit indices for the CFA and ESEM models of the CFS. Neither the two- nor the three-factor CFA models fitted the data adequately in accordance with the conventional cutoff criteria (CFI < .95, TLI < .95, and RMSEA > .10). Specification of a residual correlation (modification index = 96.4, standardized expected parameter change = .40) between item 6 (“less strength in muscles”) and item 7 (“feel weak”) improved the model fit. However, the revised three-factor CFA model still failed to provide an acceptable fit.

**Table 2:** Model fit for the CFA and ESEM models of the CFS

Model	*χ* ^*2*^	df	CFI	TLI	RMSEA (90 % CI)	SRMR	BIC
CFA							
two-factor	823.2**	43	.863	.824	120 (.113–.127)	.068	30,331.9
three-factor	569.8**	41	.907	.875	101 (.094–.109)	.061	30,034.3
Revised three-factor	482.5**	40	.922	.893	094 (.086–.101)	.059	29,938.3
ESEM							
two-factor	491.3**	34	.919	.870	103 (.095–.112)	.039	30,018.5
three-factor	263.6**	25	.958	.908	087 (.078–.097)	.024	29,761.1
Revised three-factor	138.7**	24	.980	.954	062 (.052–.072)	.018	29,656.1
ESEM + covariates	228.5**	64	.974	.956	045 (.039–.052)	.018	29,632.1

*df* degree of freedom, *CFI* comparative fit index, *TLI* Tucker–Lewis index, *RMSEA* root mean square error of approximation, *SRMR* standardized root mean square residual, *BIC* Bayesian information criterion, ** *p* < .01

Regarding the ESEM models, the two-factor model provided a mediocre fit to the data. The revised three-factor model, which specified a residual correlation between items 6 and 7 (modification index = 118.6, standardized expected parameter change = .36), showed an adequate fit to the data (CFI and TLI > .95, RMSEA ~ .06, and SRMR < .02). It also had a substantially lower BIC than the other models. For the four-factor ESEM model, the fourth factor had only one practically significant loading on the items, thus exhibiting little incremental value over the three-factor model. Overall, these findings supported the three-factor ESEM model and we further explored the reliability and convergent validity of the three CFS factors.

The factor loading matrix of the revised three-factor ESEM model is shown in Table 1. The first factor corresponded to physical fatigue (*α* = .80) and had practically significant loadings on the first three items (*λ* = .44–.87). The second factor loaded practically significantly onto items 4–8 (*λ* = .43–.97) and measured low energy (*α* = .82). The third factor referred to mental fatigue (*α* = .86) and showed practically significant loadings on the last four items (*λ* = .46–.88). The correlations among the ESEM factors ranged from .33 to .74, compared with .50–.78 for the CFA factors.

### Convergent validity

**Fig. 1:**
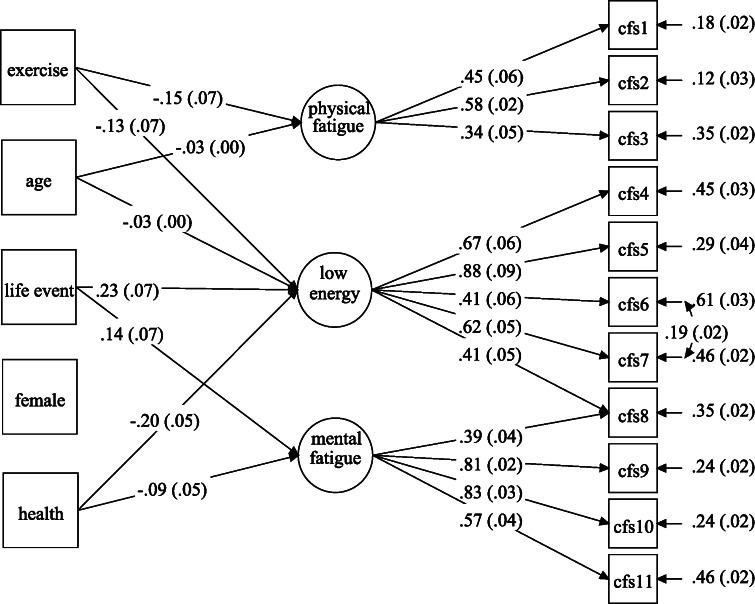
Associations between the CFS factors and covariates in the ESEM model

The ESEM model with covariates provided an adequate fit to the data (CFI and TLI > .95, RMSEA < .06, and SRMR < .02) and a substantially lower BIC. The associations between the CFS factors and the covariates are presented in Fig. 1. Participants who were older or engaged in regular exercise reported significantly lower levels of physical fatigue and higher energy. Those with worse perceived health or experience of a major life event reported significantly higher mental fatigue and lower energy.

Table 3 presents the correlations for the CFS factors with concurrent health outcomes. All three factors were positively and moderately correlated with anxiety (*r* = .32–.47, *p* < .01), depression (*r* = .31–.50, *p* < .01), and exhaustion (*r* = .41–.59, *p* < .01) and weakly correlated with sleep disturbance (*r* = .21–.30, *p* < .01). Higher levels of the three fatigue factors were modestly associated with a poorer physical quality of life (*r* = −.20 to −.23, *p* < .01) and moderately associated with a poorer mental quality of life (*r* = −.33 to −.53, *p* < .01).

**Table 3:** Correlations between the CFS factors and concurrent health outcomes

Outcome	Physical fatigue	Low energy	Mental fatigue
Anxiety	.32**	.47**	.46**
Depression	.31**	.50**	.42**
Exhaustion	.41**	.59**	.42**
Sleep disturbance	.21**	.30**	.25**
Physical quality of life	−.21**	−.23**	−.20**
Mental quality of life	−.33**	−.53**	−.42**

** *p* < .01

## Discussion

This study re-examined the factor structure and convergent validity of the CFS using ESEM in a large sample of 1259 Chinese community-dwelling adults. In contrast to the original two-factor structure [4], our results support a three-factor structure consisting of physical fatigue, low energy, and mental fatigue. The discrepancy can be attributed to the methodological inadequacies of previous validation studies and differences in analytic methods (exploratory factor modeling versus principal component analysis and oblique geomin rotation versus orthogonal varimax rotation). Rather than relying on the problematic eigenvalue > 1 criterion to determine the number of factors, we systematically compared the model fit of both two- and three-factor models, finding the three-factor model to outperform the two-factor model in both CFA and ESEM.

The revised three-factor ESEM model provided a good fit to the data and had the lowest BIC of any of the models. Except for item 8 (“hard to concentrate”), all CFS items had practically significant loadings on exactly one factor. The residual correlation specified between item 6 (“less strength in muscles”) and item 7 (“feel weak”) likely reflects the substantial overlap in the two items’ content. Satisfactory reliability and moderate to strong correlations were found among the CFS factors, suggesting adequate discriminant validity.

The ESEM model with covariates provided some substantively interesting results and supported good convergent validity for the CFS. Higher levels of fatigue were linked to greater psychological and physical distress and a poorer quality of life. In general, the results match with the findings of previous studies [8, 13]. Future longitudinal studies are needed to elucidate the causal pathways and predictive validity of fatigue on physical and mental outcomes. Although participants’ fatigue levels did not differ significantly across gender, age and regular exercise appeared to be significant predictors of lower physical fatigue and higher energy. Similarly, a poor self-perception of health and recent experience of a major life event were significantly associated with greater mental fatigue. Further studies should attempt to delineate the profile of and identify individuals with heightened fatigue levels via mixed modeling techniques [29]. Such research could in turn enable early intervention to alleviate the fatigue symptoms of these individuals.

From an analytical perspective, the significant and substantial interfactor correlations we found support the use of oblique geomin rotation rather than orthogonal varimax rotation to avoid distortion of the factor structure. Consistent with the findings of recent studies [17, 30], we found the ESEM models to provide a better fit to the data than traditional CFA models. The use of the BIC, which avoids model over-fitting by imposing penalties on the number of model parameters, substantially favored the ESEM models over the CFA models. The ESEM solutions resulted in reduced interfactor correlations, suggesting that ESEM factors are more distinct and less susceptible to multicollinearity problems. We conclude that ESEM is a helpful tool for model building and development [18] and recommend that future psychometric studies abandon the use of outdated methods and apply ESEM as a practical alternative to CFA in assessing the psychometric properties of scales.
